# Paracolic Gutter Routing: A Novel Retroperitoneal Extra-Anatomical Repair for Infected Aorto-Iliac Axis †

**DOI:** 10.3390/jcm12175765

**Published:** 2023-09-04

**Authors:** Hazem El Beyrouti, Mohamed Omar, Cristi-Teodor Calimanescu, Hendrik Treede, Nancy Halloum

**Affiliations:** 1 Department of Cardiac and Vascular Surgery, University Medical Center, Johannes Gutenberg University, 55131 Mainz, Germany; 2 Division of Vascular Surgery, Ameos Clinic Center Bremerhaven, 27568 Bremerhaven, Germany

**Keywords:** aorta, infection, graft, prosthesis, vascular, endovascular, VGEI, INAA

## Abstract

Objective: We describe and analyze outcomes of a novel extra-anatomical paracolic gutter routing technique for surgical repair of aorto-iliac infections. Methods: A double-center, observational, cohort study of all consecutive patients with aorto-iliac infections treated using extra-anatomical paracolic gutter technique. Between May 2015 and December 2022, six patients with aorto-iliac infections were treated with the paracolic gutter routing technique. Cases were identified retrospectively in an institutional database, and data were retrieved from surgical records, imaging studies, and follow-up records. Results: Aorto-bifemoral vascular reconstructions were performed using this technique in six patients. During mean follow-up of 52 ± 44 months, there was one case of graft thrombosis (17%) with subsequent successful thrombectomy. Primary and secondary graft patency rates were 83% and 100%, respectively. There was one mortality (17%) due to candida sepsis. All graft prostheses were patent at last follow-up. Conclusions: The paracolic gutter technique is a useful technique in patients with extensive aorto-iliac infections, arteriovenous and iliac-ureteric fistulas, or at a high risk of vascular graft infection and is associated with favorable reinfection and patency rates.

## 1. Introduction

Graft infections are infrequent (incidence 0.6–5%) but serious complications of vascular surgery. Aorto-iliac graft infections can threaten limb viability and are associated with high morbidity and mortality rates. A conservative treatment is associated with high mortality rates of 25–88% and limb loss in 5–25% [1,2,3]. The mortality and morbidity of aortic infection depend on several factors, including the presence of concomitant comorbidities, the causative agent, the invasivity of the infection, the timing of diagnosis, and the operative management [4,5].

In one study of 72 vascular graft infections (VGI) with positive graft cultures in 65 patients, infection-related mortality was 11%; of the 65 patients, 14 had undergone aorto-bifemoral bypass, 13 axillo-femoral bypass, 5 femoro-femoral bypass, 27 femoro-popliteal bypass, and 4 femoral endarterectomy with synthetic patch angioplasty [6]. Surgical debridement and replacement of infected segments is necessary, but the close proximity of synthetic grafts to the infected field may lead to high rates of reinfection. Several graft materials for in situ reconstruction (using an antibiotic soaked or rifampicin-bounded graft, cryopreserved allograft, autogenous femoral vein, tube graft made of bovine pericardium, silver-coated prostheses, Omniflow or cryopreserved allograft) have been studied to prevent infection recurrence with variable results [7,8,9,10,11,12,13,14,15,16]. Extra-anatomical bypass techniques (axillo-femoral bypass) have also been proposed but with increased morbidity: low patency (40–73% at 5 years), limb amputation (up to 24%), aortic stump blow-out (2–30%), and mortality (up to 27%) [11]. None of these approaches is universally applicable due to sometimes there being longer graft preparation times, the limited availability of certain prostheses, and increased risk of reinfection. We describe here a novel extra-anatomical para-colic technique to manage aorto-iliac infections and clinical outcomes of patients treated to date with this technique.

## 2. Methods

This is a retrospective review of all consecutive patients who received extra-anatomical paracolic gutter technique as aorto-bifemoral grafts. Cases were identified retrospectively in our institutional database, and data were retrieved from surgical records, imaging studies, and follow-up records. Medical records of all patients with confirmed infection between May 2015 and December 2022 were screened for inclusion in the study. Patients treated using other surgical techniques, with incomplete medical records or who did not meet the diagnostic criteria for VGI were excluded from the analysis. Demographic, clinical, and laboratory data were collected from electronic medical records. Variables of interest included age, sex, and underlying medical conditions.

The choice of technique was based mainly on the preference of the operating surgeon. In general, this technique was used in patients with aorto-iliac infections. Infection diagnosis was established by computed tomography (CT) supported by clinical, microbiological, and laboratory findings, and was confirmed by intraoperative findings.

Ethical approval was obtained from the institutional ethical committee, and informed consents were waived due to the retrospective and observational nature of this study. 

The outcomes analyzed distinguished between early (≤30 days after graft placement) and late (>30 days) surgical revision, primary and secondary patency and early and late mortality, reinfection, and freedom from formation of aorto-enteric or aorto-ureteral fistulation during the follow-up after vascular reconstruction using the novel extra-anatomical paracolic gutter routing technique in aorto-iliac infection.

### 2.1. Surgical Technique

Prior to surgery, all patients undergo a thorough preoperative evaluation, which includes a detailed medical history, physical examination, and infectious disease consultation. Typical imaging studies such as CT angiography are performed to assess the extent of infection, anatomical considerations, and the feasibility of the extra-anatomical repair approach. In cases of sepsis and hemodynamic instability, patients are admitted to intensive care for stabilization prior to surgery.

Bilateral ureteral splints are inserted 1–2 days before surgery. The extra-anatomical aorto-bifemoral graft procedure is carried out under general anesthesia and through a midline laparotomy with the patient in the supine position. The abdomen is explored to assess the extent of infection and to identify any intra-abdominal involvement.

To facilitate exposure, we use a self-retaining retractor to retract the omentum and transverse colon cephalad. The small bowel is removed laterally. We irrigate the infected area with an antibiotic solution, which is chosen on the basis of the pre-operative cultures. Our routine intraoperative anticoagulation regimen includes unfractionated heparin prior to arterial clamping (5000 IE).

**Figure 1:**
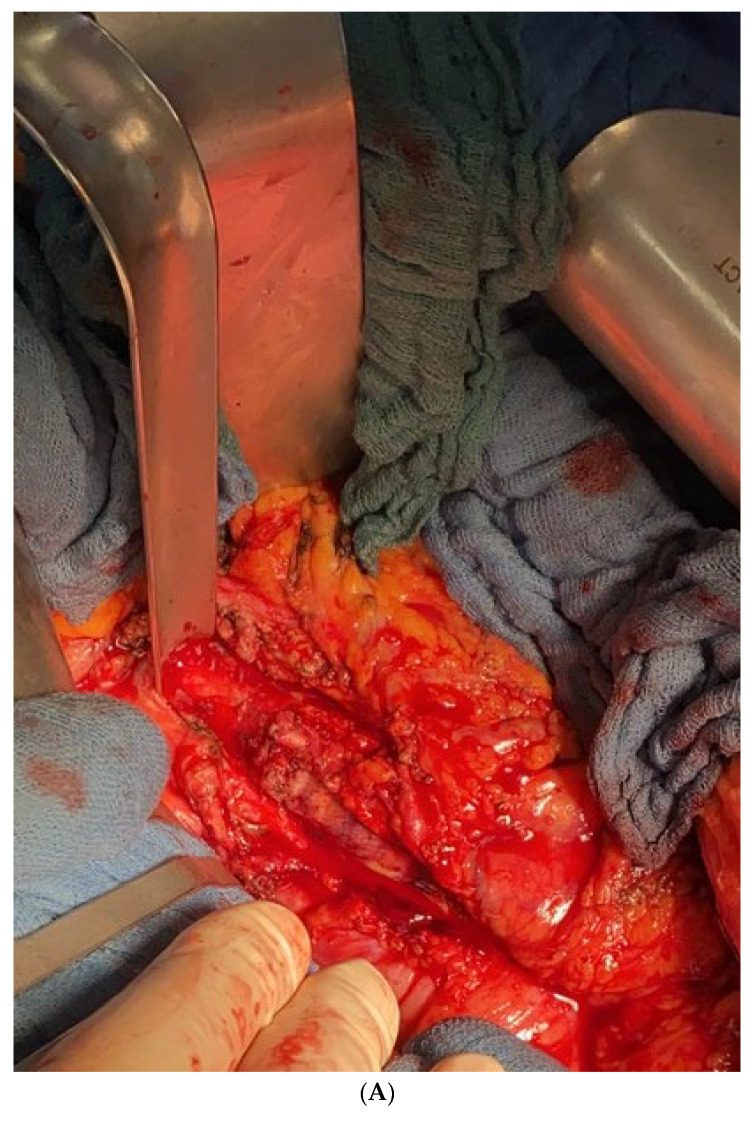

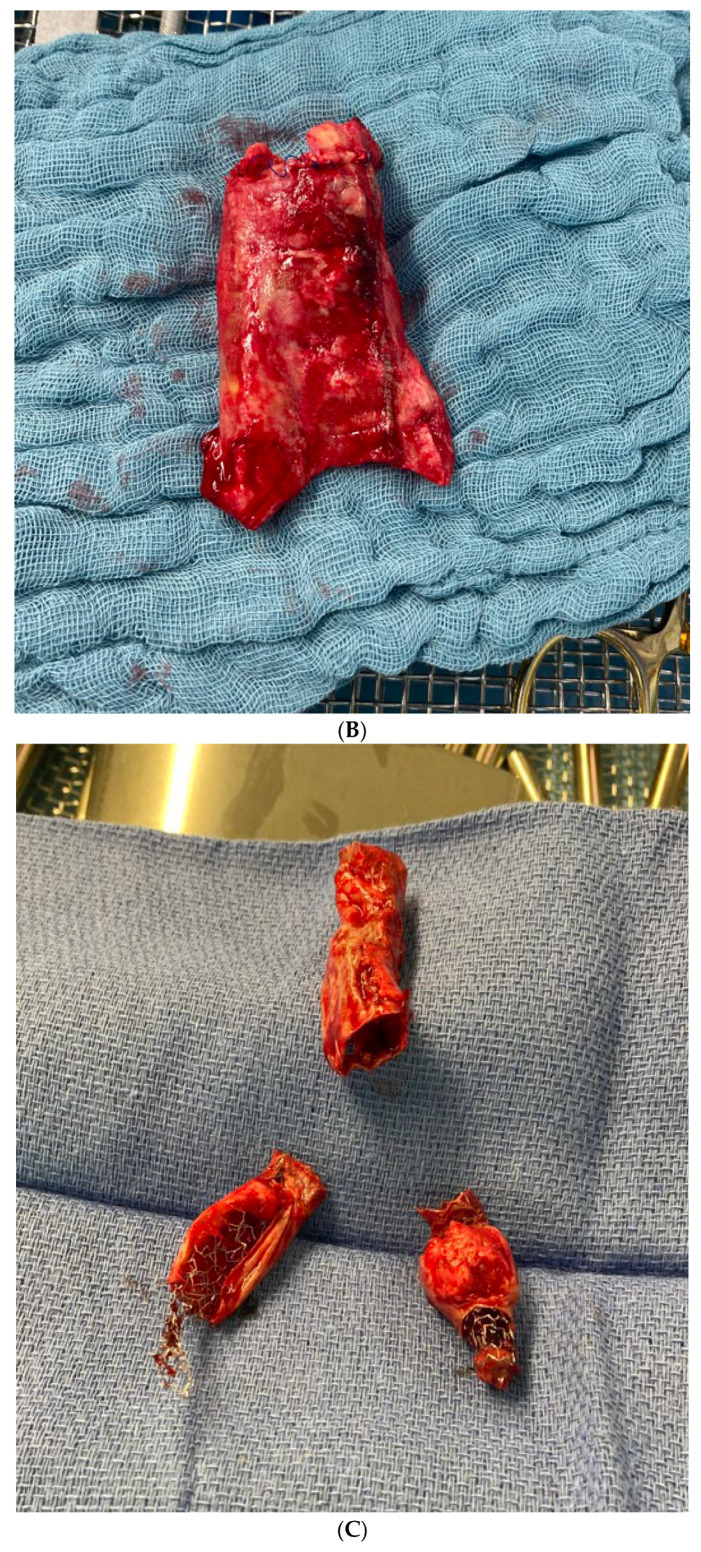
(**A**) Infection of bovine pericardial reconstruction and stents in the aorto-iliac axis by redo aorto-axis reconstruction in a patient operated four times previously. (**B**) The infected pericardial tube is resected. (**C**) Removal of infected implanted stents in the aorto-iliac axis.

**Figure 2:**
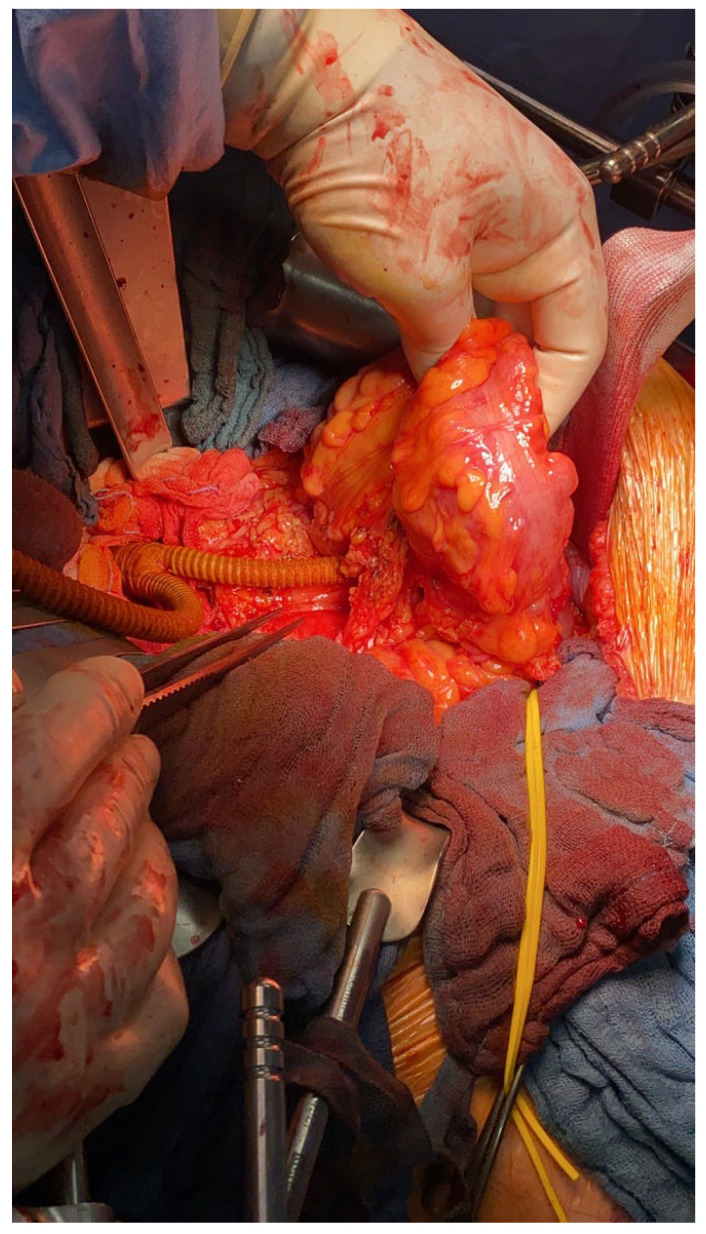
The branch of the vascular graft on each side is passed retrocolon and retro-peritoneally through the channel and laterally into the paracolic gutter.

**Figure 3:**
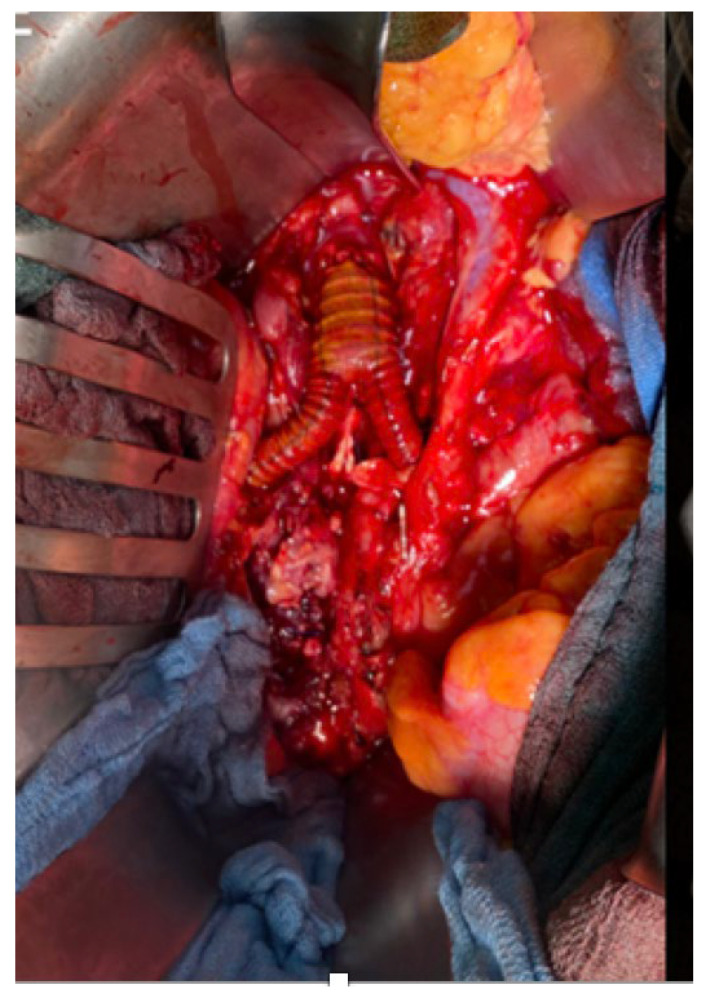
Intraoperative replacement of infrarenal aorta with a silver–triclosan collagen-coated polyester vascular graft.

**Figure 4:**
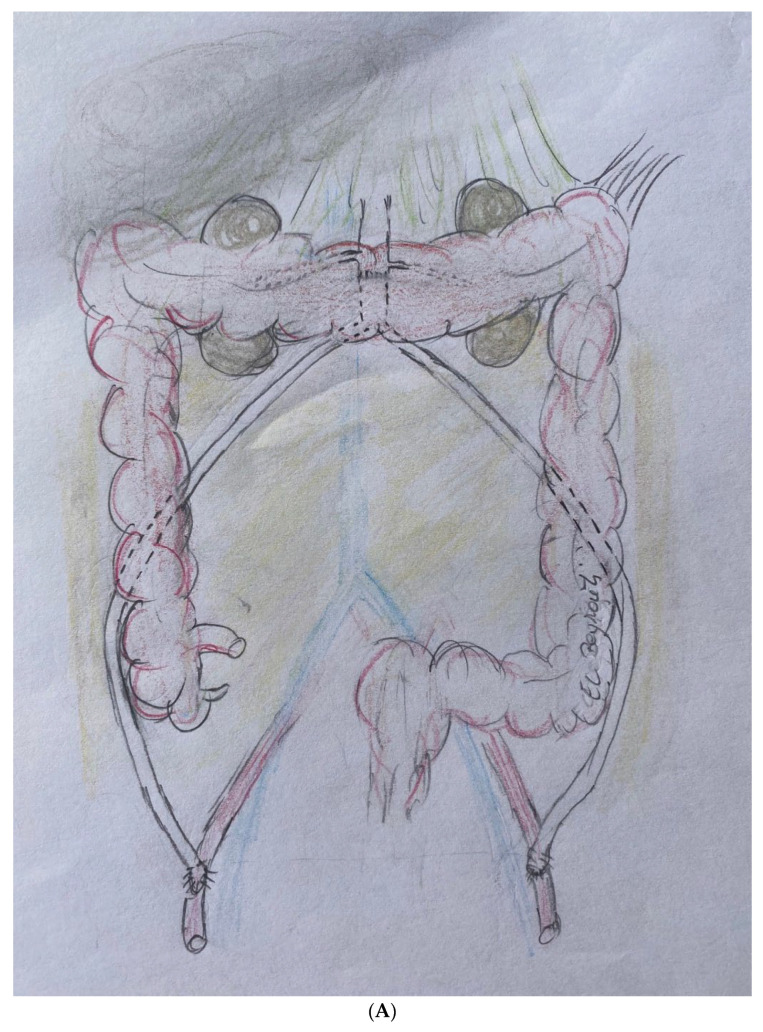

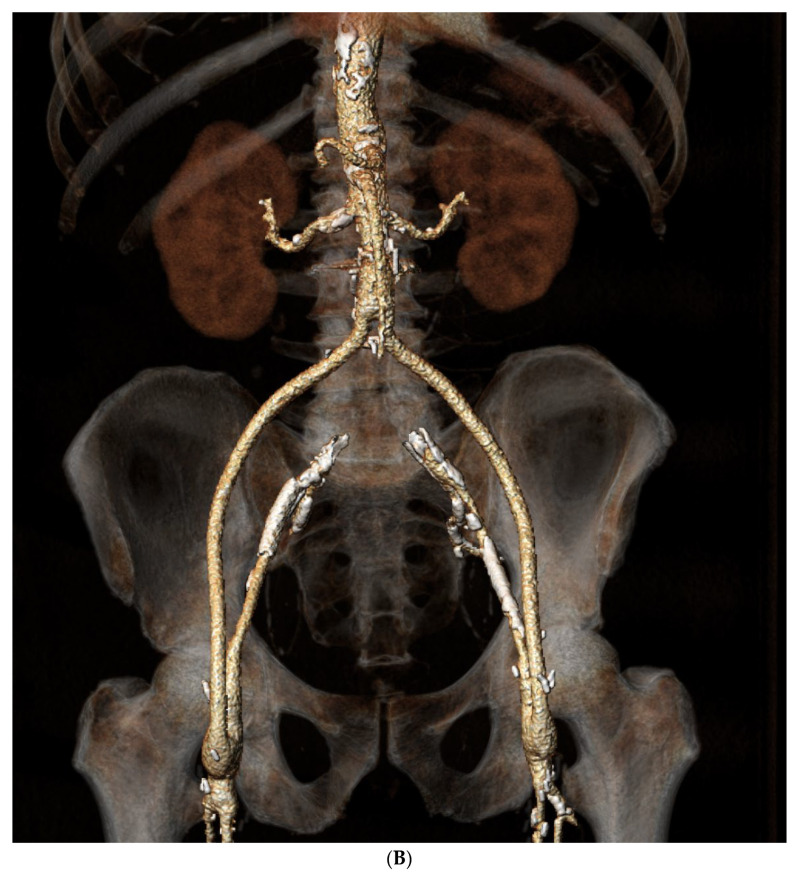
(**A**) Artistic representation of the technique. (**B**) Postoperative, volume-rendering three-dimensional computed tomographic reconstruction of the aorto-iliac grafts with para-colic routing.

The infected aorto-iliac axis is exposed by meticulous dissection and isolation of the vascular structures. Proximal vascular control is achieved using vascular clamps and the iliac vessels are occluded with Foley catheters. After cross clamping the aorta and the iliac vessels, all infected native and synthetic materials are removed (Figure 1B,C), and surrounding tissues are radically debrided. The proximal segment of the aorta is anastomosed in an end-to-end fashion with 4–0 Prolene running sutures to either an antibiotic-soaked Dacron Y-graft or a silver-triclosan collagen-coated polyester graft selected based on anatomical considerations and availability. A new channel is created behind the ureter (which can be easily palpated using the ureteral splint) and the colon on either side using blind separation. The extra-anatomical bypass is performed by creating tunnels through the retroperitoneal space, carefully dissected to avoid injury to vital structures. The branch vascular graft on each side is then passed retroperitoneally through the channel and laterally into the paracolic gutter (Figure 2), and then extended along the lateral abdomen wall to the groin, where it is anastomosed to the femoral artery using 4–0 Prolene running sutures (Figure 3 and Figure 4A). Complete retro-peritonealization of the graft is thus ensured. The proximal and distal ends of the graft are anastomosed to the uninfected portions of the aorta and femoral vessels using standard vascular techniques. An omental flap is added unless the omentum is too small to cover the reconstruction. Care should be taken to ensure that the graft is not subjected to excessive tension or kinking. The retroperitoneal tunnels are closed using absorbable sutures (Video S1).

Postoperatively, we recommend prophylactic heparin and aspirin 100 mg once daily during hospital stay if there are no other indications. All patients are closely monitored in intensive care.

### 2.2. Intraoperative Microbiological Cultures and Antimicrobial Therapy

Accurate diagnosis of VGI can involve several radiological and nuclear medicine modalities with white blood cell scintigraphy or PET recommended to improve diagnostic accuracy [17]. Our standard practice is to request for PET-CT unless the patient is considered at risk of a severe complication like sepsis. Specimens collected intraoperatively are routinely sent for culture; in the case of prosthetic graft infections; a piece of the removed graft is also sent for microbiological testing. Infection is classification according to the Management of Aortic Graft Infection Collaboration (MAGIC) criteria [18]. All patients receive antifungal or wide-spectrum antibiotic intravenous therapy upon diagnosis—subsequently adapted according to the pathogens identified from intraoperative microbiological cultures—and is continued for a minimum of six weeks after discharge and as per laboratory markers and microbiological findings of infection. In the cases of candida infection, we typically advise antibiotic medication for at least one year. Graft-related outcomes such as patency are evaluated during follow-up using ultrasonography and/or CT.

### 2.3. Statistical Analyses

Descriptive statistics are used to summarize patient demographics, intraoperative variables, and postoperative outcomes. Statistical Package of Social Sciences for Windows, version 20 (SPSS Inc., Chicago, IL, USA) was used for statistical analysis. Continuous numerical variables are presented as median  ± standard deviation and were compared with the Student’s *t*-test. Categorical variables are presented as numbers and percentages and were compared with a chi-square test.

## 3. Results

Six patients with aorto-iliac infections were treated using the paracolic gutter technique (Table 1, Figure 1A). At the time of presentation, the average age was 69 ± 12 years. The cohort included three men and three women. Mean BMI was 27 ± 4 kg/m^2^. Comorbidities included the following: six (100%) had chronic obstructive pulmonary disease (COPD) and hypertension, five (83%) had hyperlipidemia and were smokers at the time of surgery, four (67%) had diabetes mellitus, malignancy, and peripheral artery disease, and three (50%) had coronary artery disease. One (17%) patient had undergone prior endovascular repair, two (33%) patients had recurrent reinfections, three (50%) patients had undergone at least one redo procedure, and four (67%) had contained rupture at the aorto-iliac axis. Infective native aortic aneurysms were present in two (33%) patients, and an aorto-ureteric fistula was present in one (17%) patient. (Table 2).

Operative and procedural details are given in Table 2. All patients were confirmed with a major infection according to the MAGIC classification: four with VGI and two with infective native aortic aneurysm. The following bacteria were detected microbiologically from positive intraoperative cultures in all six (100%) patients: Staphylococcus and Enterococcus (34% each); Pseudomonas and Candida (17% each). 

All procedures were technical successes and there were no major intraoperative or early postoperative complications. No intraoperative deaths occurred. Median procedure time was 322 ± 44 min. The median length of intensive care stay was 2.2 ± 1 days and total hospitalization was 17 ± 6 days. 

Graft patency was assessed through regular postoperative imaging. There was no early surgical revision and one late (>30 days) due to graft thrombosis (treated with thrombectomy). Primary and secondary graft patency rates were 83% and 100%, respectively. Postoperative infection control was evaluated based on clinical and radiological parameters. All six patients demonstrated complete resolution of infection as evidenced by the absence of fever, wound discharge, and negative imaging findings for infection at discharge. There were no signs of aortic infection with formation of aorto-enteric or aorto-ureteral fistulation (Figure 4B). No complications were observed. Patients were followed for median 52 ± 44 months. One late mortality occurred due to candida reinfection with sepsis and respiratory failure, and to multiorgan failure. This patient had been advised to continue long-term antimycotic therapy but was subsequently discovered to have discontinued medication eight months previously of his own accord.

During the first six postoperative months, all patients received dual antiplatelet therapy (aspirin, clopidogrel). Three (50%) continued with dual antiplatelet therapy due to coronary artery stenting for coronary artery disease or atrial fibrillation. Three (50%) switched to aspirin alone.

## 4. Discussion

Infections of the aorto-iliac bifurcation—especially fungal infections and those involving arteriovenous and iliac-ureteric (IUF) and iliac-duodenal (IDF) fistulas—are rare but very challenging surgical problems to address. Aorto-iliac infection has a mortality rate between 9% and 75% and associated morbidity can include amputation (up to 30%) depending on the severity of the infection and type of treatment [19,20,21]. Those that occur after previous aortic surgery are often more difficult to treat and have a higher mortality rate due to the presence of scar tissue from the previous surgery. Reinfection in the aortoiliac arteries can cause graft occlusion, sepsis, limb ischemia, internal organ ischemia, sepsis, and aortic rupture. 

Several techniques have been described for the treatment of aortic graft infections, ranging from isolated anti-infection therapy to graft explantation with extensive debridement of all infected tissue followed by revascularization using extra-anatomic bypass or in situ reconstruction [9,11,12,13,14,15,16]. All these approaches remain challenging because of prolonged operating times, sufficient length of the autologous graft, limited availability of certain prostheses, patency (especially through extra-anatomical bypass), and the risk of re-infection [9,22]. In a study of 122 VGI patients with various surgical approaches—semiconservative (21%) with infection drainage and preservation of the vascular prosthesis; resection (38%) with extra-anatomic bypass; and in situ reconstruction (32%)—the semi-conservative approach was associated with the poorest long-term outcomes [23]. 

The proximity of the graft to the infected area is associated with a high rate of infection, which is why many surgeons tend to perform an extra-anatomic bypass [24]. But disappointing results of the extra-anatomic bypass due to high mortality (up to 27%), poor patency rates (40–73% at 5 years), high amputation rates (up to 24%), and risk of aortic stump rupture (2–30%) [11] have encouraged many to resort to in situ reconstruction with autogenous grafts. However, the results of autologous reconstruction with femoral vein (FV) were not encouraging due to operative mortality (up to 10%) and low survival (45% at five years); in addition the venous morbidity after FV harvest was up to 14% of deep venous thrombosis [25].

Fistulas between aortic graft and the adjacent ureter or duodenum can lead to severe morbidity and mortality if untreated. Endovascular treatment of IUF and IDF has emerged as a promising alternative to open surgical repair, offering a less invasive and potentially safer option by placing a stent-graft over the fistula, effectively sealing the abnormal junction between the aortic graft and the adjacent ureter or duodenum [26]. The literature includes several case reports with high rates of technical success and favorable outcomes with endovascular repair of IUF and IDF [26,27,28]. 

However, the endovascular option for IDF remains technically challenging and requires careful stent-graft selection, prudent deployment, and close postoperative monitoring for stent-graft-related complications. In addition to these technical challenges, endovascular repair is associated with several potential complications, including stent-graft migration, endoleaks, infection, bleeding, puncture site complications, and thrombosis and thromboembolic events [29,30]. Furthermore, long-term outcomes have not been established.

A methodological review of 245 reports (445 patients with arterio-ureteral fistula) showed that the predominant location of the fistula was the common iliac artery and that mortality ranged from 7–19% [31]. In recent years, the treatment of aorto-iliac infection has shifted from open surgical repair to minimally invasive endovascular stenting. Because most surgeons have treated only isolated cases, treatment algorithms are often ill-defined, especially in the case of arterio-ureteral or arterio-duodenal fistula. This is because these patients usually have a challenging environment due to previous extensive pelvic surgery and radiation therapy causing adhesions and fibrosis. Endovascular surgery is preferred over open surgery because of improved arterio-ureteral fistula related mortality (4% vs. 11%) [26,31].

Our experience indicates that the paracolic gutter course is an excellent alternative. This approach avoids the proximity of graft materials to the infected area and permits intra-abdominal and juxta-anatomic implantation, which is preferable to the extra-anatomical subcutaneous course of the axillo-bifemoral bypass. Moreover, the channel-in-channel course permits omental flap covering and complete retro-peritonealization. Another advantage of this approach is that it is feasible in patients with large aortas where mismatch between the aorta and certain prostheses (such as femoral venous or biosynthetic graft prostheses) might be an issue. Ureteral splints implanted pre-operatively facilitate the procedure considerably and ensure recto-colonic and retro-ureteral routing and minimize the risk of iatrogenic injury.

The limitations of this study can be seen in the retrospective nature of the analysis, the small number of patients, and the limited follow-up period. However, such series are necessary to describe experience with these relatively rare cases and to share novel techniques.

## 5. Conclusions

Creation of a neo-aortofemoral system using the retro-peritoneal paracolic gutter is a safe approach in a heterogeneous cohort of patients with aorto-iliac graft infections and presents an alternative to other methods that can be associated with a high risk of re-infection. Long-term follow-up with imaging and to ensure medication compliance is required.

## Figures and Tables

**Table 1:** Summary of patient data.

	*n* = 6
Male	3 (50%)
Age (years)	69 ± 12
Peripheral artery disease	4 (67%)
BMI (kg/m^2^)	27 ± 4
Coronary artery disease	3 (50%)
Hypertension	6 (100%)
Hyperlipidemia	5 (83%)
Diabetes	4 (67%)
Smoking	5 (83%)
COPD	6 (100%)
Malignancy	4 (67%)
Prior endovascular repair	1 (17%)
Aorto-iliac rupture	4 (67%)
Recurrent infections	2 (33%)
Prior tumor resection	3 (50%)
Infective native aortic aneurysm	2 (33%)
Aorto-ureteric fistula	1 (17%)
ASA ≥ 4	5 (83%)

Data are *n* (%) or (median ± SD). ASA, American Society of Anesthesiologists; BMI, body mass index; COPD, chronic obstructive pulmonary disease; SD, standard deviation.

**Table 2:** Indication and operative data.

	*n* = 6
Infective native aortic aneurysm (INAA)	2 (33%)
Vascular graft/endograft infection (VGEI)	4 (67%)
Major infection (MAGIC classification)	6 (100%)
Antibiotic-soaked Y-graft (Dacron)	3 (50%)
Silver–triclosan collagen-coated (polyester)	3 (50%)
Omental flap	3 (50%)
Procedure time (min)	322 ± 44
Technical success	6 (100%)
Procedural complications	0
Perioperative mortality	0
Intensive care (days)	2.2 ± 1
Hospitalization (days)	17 ± 6
Positive cultures/Infection (Intraoperative microbiological cultures)	
*Staphyolococcus*	2 (34%)
*Enterococcus*	2 (34%)
*Pseudomonas*	1 (17%)
*Candida*	1 (17%)

Data are *n* (%) or (median ± SD). MAGIC, Management of Aortic Graft Infection; SD, standard deviation; INAA, Infective native aortic aneurysm; VGEI Vascular graft/endograft infection.

## Data Availability

The data presented in this study are available on request from the corresponding author.

## Supplementary Materials

The following supporting information can be downloaded at: https://www.mdpi.com/article/10.3390/jcm12175765/s1, Video S1: Paracolic gutter routing.
